# Supplementary figures and images for: Mycorrhization of Quercus acutissima with Chinese black truffle significantly altered the host physiology and root-associated microbiomes
Source: PeerJ. 2019 Feb 18;7:e6421. doi: 10.7717/peerj.6421 (PMC6383558; doi:10.7717/peerj.6421)

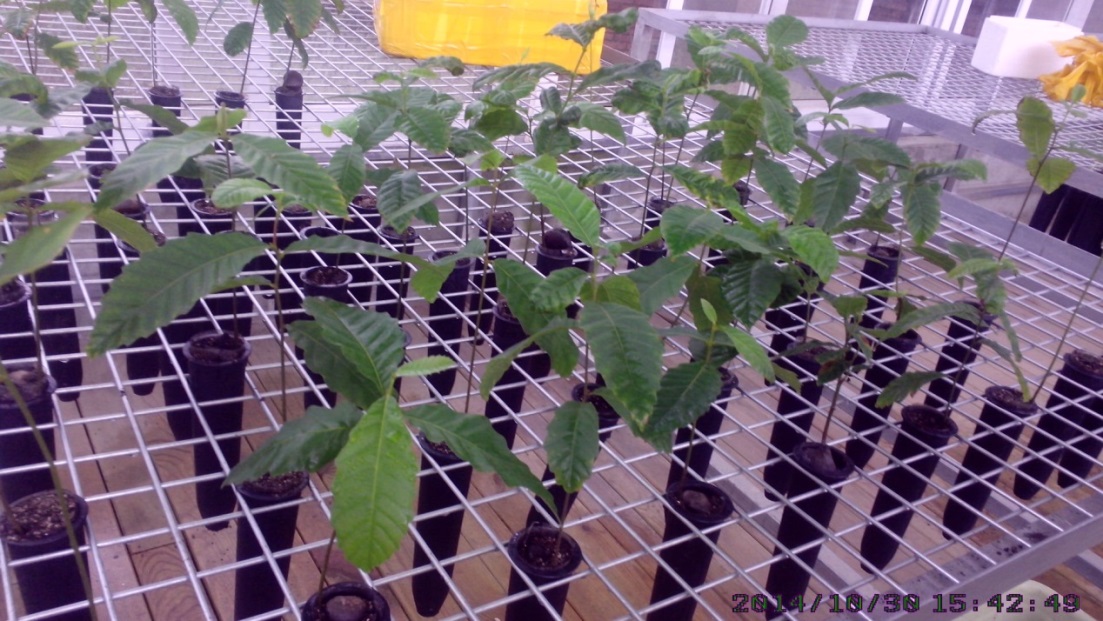

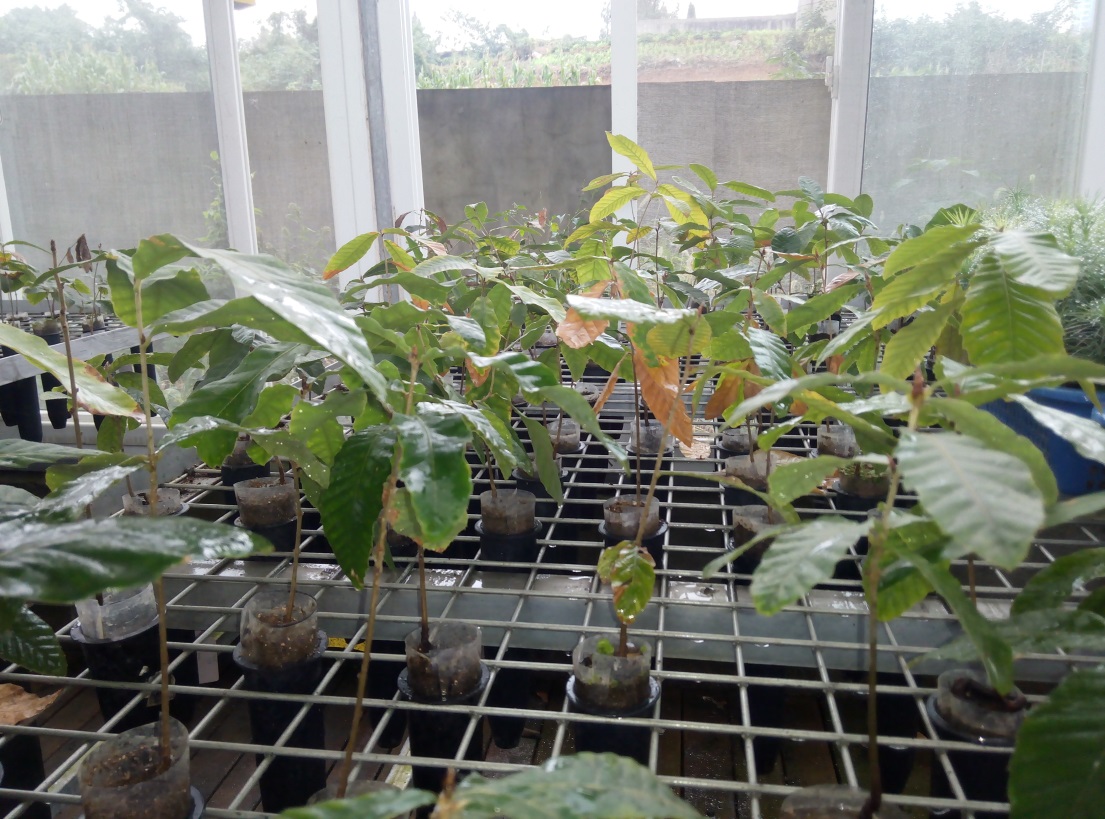

Supplement: File S1 — The growth time we investigated was from March to November. The water content in the substrate was about 50%. From March to July, the average temperature was 22.2 °C (10.7–35.6 °C) and the average humidity was 78.8% (45–100%). From July to November, the average temperature was 24.0 °C (12.7–38.7 °C) and the average humidity was 82.07% (39–100%). Photo credit: Lei Ye. [file peerj-07-6421-s001.docx]

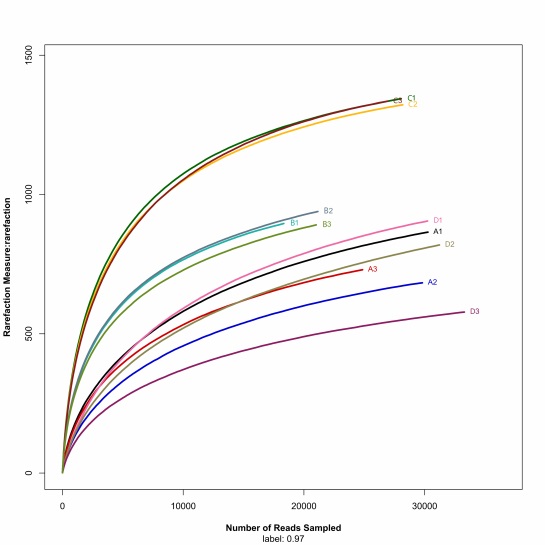

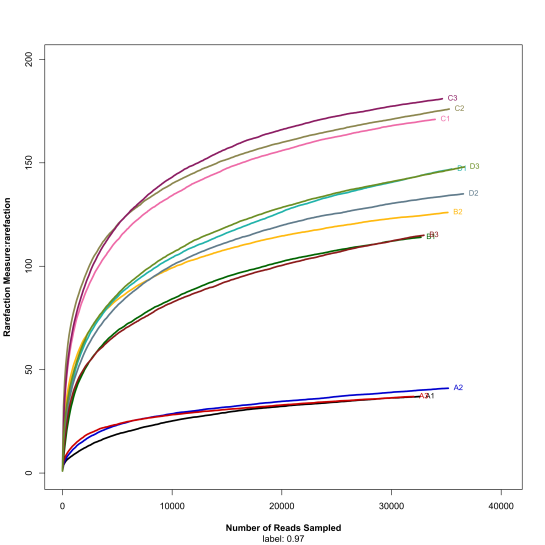


**a b**

Supplement: File S2 — (A), ectomycorrhizae from Q. acutissima mycorrhized with T. indicum. (B), the ectomycorrhizosphere soil of Q. acutissima. (C), the rhizosphere soil without T. indicum associations (control soil). (D), roots from cultivated Q. acutissima without T. indicum colonization (control roots). [file peerj-07-6421-s002.docx]

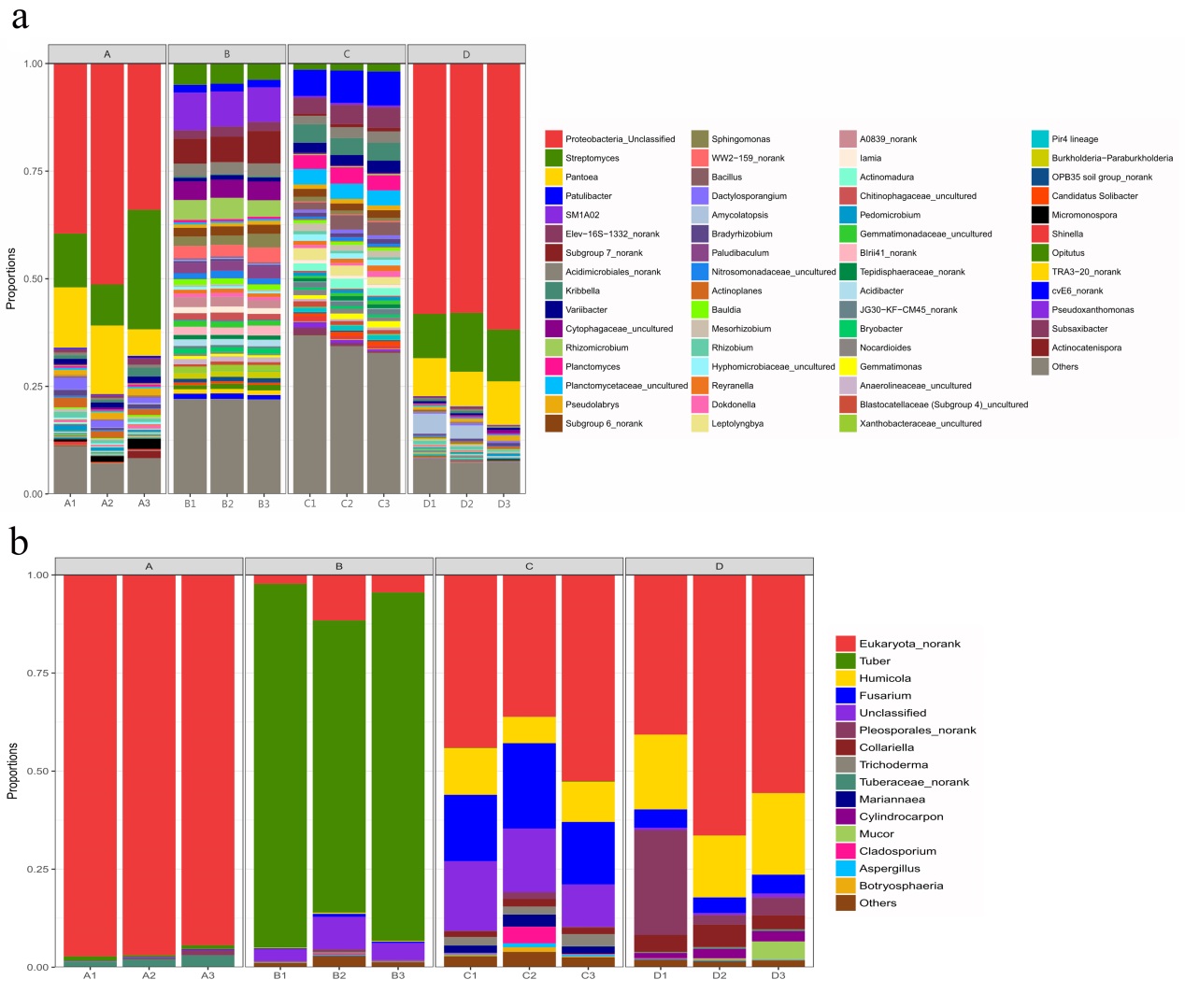

Supplement: File S3 — (A), ectomycorrhizae from Q. acutissima mycorrhized with T. indicum. (B), the ectomycorrhizosphere soil of Q. acutissima. (C), the rhizosphere soil without T. indicum associations (control soil). D, roots from cultivated Q. acutissima without T. indicum colonization (control roots). “norank” means there is no scientific name for this hierarchy in the taxonomic family tree. “Unclassified” means under the confidence threshold, it cannot be compared with the database. All experiments were conducted with three replicates. [file peerj-07-6421-s003.docx]

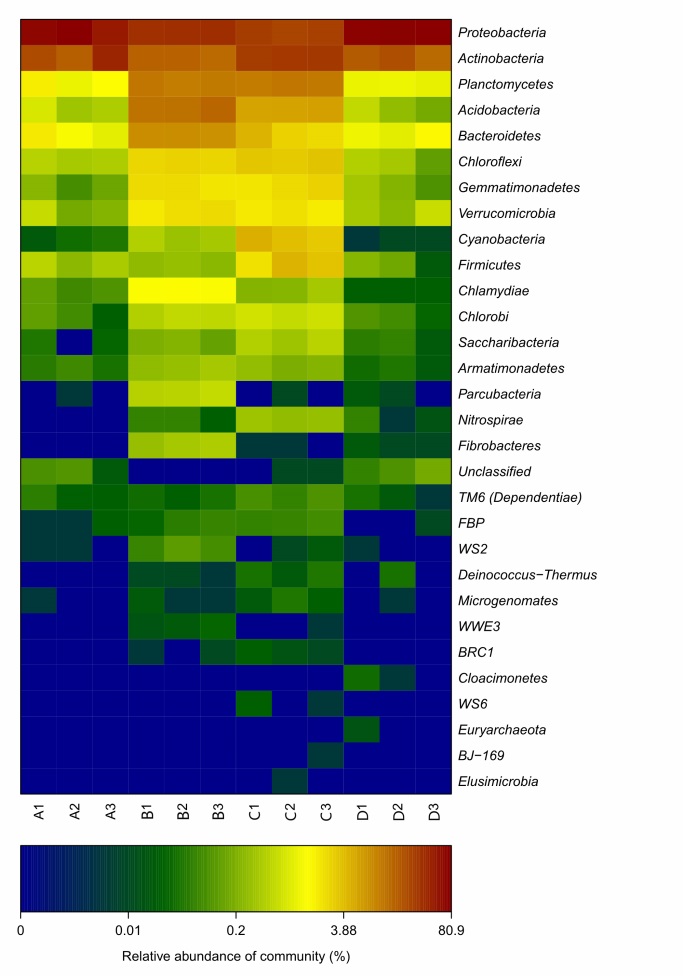

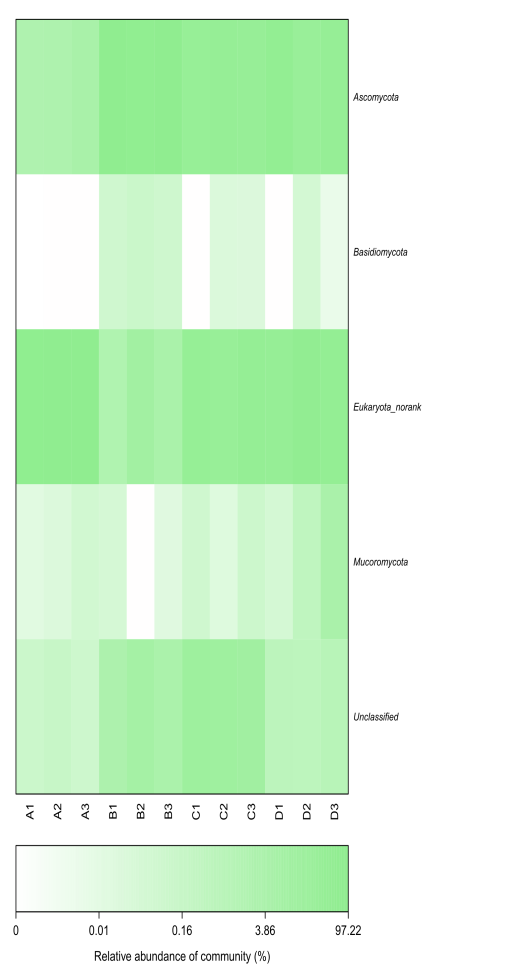


**a b**

Supplement: File S4 — (A), ectomycorrhizae from Q. acutissima mycorrhized with T. indicum. (B), the ectomycorrhizosphere soil of Q. acutissima. (C), the rhizosphere soil without T. indicum associations (control soil). (D), roots from cultivated Q. acutissima without T. indicum colonization (control roots). [file peerj-07-6421-s004.docx]
